# Levels of Galectin-3 in Chronic Heart Failure: A Case-Control Study

**DOI:** 10.7759/cureus.28310

**Published:** 2022-08-23

**Authors:** Altaff Khadeja Bi, Viswan Santhosh, Karthik Sigamani

**Affiliations:** 1 Clinical Biochemistry, Karpaga Vinayaga Institute of Medical Sciences and Research Center, Chengalpattu, IND; 2 Pathology, Karpaga Vinayaga Institute of Medical Sciences and Research Center, Chengalpattu, IND

**Keywords:** galectin, collagen, pathogenesis, cardiac remodeling, heart failure, biomarkers

## Abstract

Introduction

Heart failure (HF) is a progressive clinical syndrome resulting from various cardiac disorders. Galectin-3 promotes adverse cardiac remodeling leading to chronic heart failure (CHF).

Aim

To estimate the levels of galectin-3 in chronic heart failure (CHF) patients and controls and to determine the association between galectin-3 levels with age, gender, and left ventricular ejection fraction (LVEF).

Materials and methods

The levels of plasma galectin-3 were estimated in CHF patients from January 2013 to October 2013 at Rajiv Gandhi Government General Hospital, Chennai, Tamil Nadu. The study was a case-control study. A total of 55 CHF patients were recruited as cases, and 55 controls were enrolled for the study. Participants' profiles were documented, and 5 mL of blood sample was collected. Galectin-3 levels in plasma were estimated by using an enzyme-linked immunosorbent assay (ELISA). Data were analyzed using SPSS 25.0 version. Mean, SD, and percentages were used to compare the characteristics of the two groups. The student's t-test was used to compare galectin-3 levels between CHF patients and the controls. ANOVA was employed to compare galectin-3 levels in the different age groups, gender, and LVEF. The receiver operating characteristic (ROC) curve was plotted for plasma galectin-3 in CHF.

Results

In the present study, the mean age of CHF patients was 55.9±8.1 years and 54.1±9.4 years for controls. Males constituted 63.6% (n=35) and females were 36.4% (n=20) in the CHF group while 67.3% (n=37) were males and 32.7% (n=18) were females in the control group. The mean and SD for plasma galectin-3 was 9.95±2.8 ng/mL among CHF patients, while it was 4.08±1.3 ng/mL among controls (p<0.0001). As the age increased, levels of plasma galectin-3 increased in CHF patients and controls (p<0.00001). However, there was no statistical significance (p >0.05) for levels of galectin-3 among males and females in both groups. There was a highly significant difference in galectin-3 levels among cases and controls when classified into sub-groups based on their LVEF (p<0.0001). At the cut-off level of 8 ng/mL, plasma galectin-3 had a sensitivity of 92% and specificity of 71% in predicting CHF.

Conclusion

Galectin-3 helps in identifying CHF due to maladaptive remodeling of the heart. The present study concludes that estimating the plasma levels of galectin-3 is useful in diagnosing CHF.

## Introduction

Heart failure (HF) is a progressive clinical syndrome resulting from various cardiac disorders. Multiple factors are responsible for the pathogenesis of HF. Some are cardiomyocyte damage, inflammation, and neuro-hormonal activation [1]. The prevalence of HF in India was about 10 million patients based on the INDUS study in 2016 [2]. HF in the initial stage is clinically silent. However, ongoing cardiac remodeling is well documented during this phase. Clinical diagnosis of HF is challenging and has reduced diagnostic significance as the major symptoms of HF overlap with many other clinical conditions. This emphasizes the need for addressing a molecule involved in the pathogenesis rather than the symptom of the disease process.

Galectin-3, a member of the galectin family, is a vital factor in the pathophysiology of HF, chiefly due to its role in remodeling the cardiac ventricles. After a myocardial injury, inflammation is triggered, accumulating activated macrophages releasing galectin-3 [ 3,4]. Initially, galectin-3 imparts a protective role by preventing further necrosis and apoptosis of the myocardium. However, sustained inflammation increases the expression of galectin-3, which now activates the quiescent fibroblasts to secrete collagen [5, 6]. As a result, excess collagen is deposited surrounding the myocytes resulting in extensive scar tissue deposition in the cardiac ventricles. Thus, maladaptive remodeling sets in, which progressively results in chronic heart failure (CHF) [7].

Galectin-3 is thought to be a "disease mediator" instead of a "disease marker" [4]. A multi-modality approach involving specialized biomarkers is essential in CHF to optimize the treatment plan and reduce the rate of re-hospitalization and mortality. Gradual worsening of HF can be identified using galectin-3. It can be used to assess a patient's prognosis for HF treatment [8]. Elevated levels of galectin-3 increase the damage to the heart and are associated with poor prognosis. Taking into consideration the role of galectin-3 in HF, this study was planned to estimate the levels of galectin-3 in CHF patients and controls and determine the association between galectin-3 levels and age, gender, and left ventricular ejection fraction (LVEF).

## Materials and methods

Levels of galectin-3 were estimated in CHF patients between January and October 2013 at Rajiv Gandhi Government General Hospital, Chennai, Tamil Nadu, after obtaining Institutional Ethical Committee clearance (IEC Ref No. 36092012). It was a case-control study done with purposive sampling. For treatment, fifty-five CHF patients attending the cardiology out-patient department (OPD) were recruited as cases. In addition, 55 adults who attended the cardiology OPD for cardiac evaluation as a part of anesthetic fitness for non-cardiac surgeries were recruited as controls for the study. All the participants were included after informed consent for participation in the study.

Inclusion criteria

CHF patients having LVEF less than 45% by echocardiogram were enrolled as cases. Patients in this group were on treatment for more than six months with standard therapy using diuretics, angiotensin-converting enzyme (ACE) inhibitors, β-blockers, and diuretics. The control group comprised adult patients without symptoms of HF and having an LVEF of more than 60% by echocardiogram.

Exclusion criteria

Patients less than 18 years old, patients with acute CHF, renal failure (eGFR less than 90 mL/min), cirrhosis of the liver, malignancy, asthma/chronic obstructive pulmonary disease (COPD), rheumatoid arthritis, multiple myeloma, stroke, chronic dermatological disease, auto-immune disorder, thyroid dysfunction, psychiatric illness, and septicemia were identified through the history and excluded in both the case and control groups.

Participants’ profiles

Data regarding the age, gender, height (centimeters), weight (kilograms), history of smoking and alcohol intake, presence of diabetes mellitus or hypertension, and LVEF from echocardiography records were collected as participants' profiles.

Procedure

Venous blood (5 mL) was sampled from participants consulting the OPD of cardiology. The blood was aliquoted into two pre-labeled tubes. Next, 3 mL of blood was transferred into an ethylenediamine tetraacetic acid (EDTA)-coated tube. In contrast, 2 mL of blood was transferred into a plain tube and transported in an ice-lined box to the laboratory. Blood samples were centrifuged immediately (within an hour) of collection. The supernatant plasma and serum were stored in a deep freezer (-70 C) until further processing.

Serum creatinine was estimated for all the participants by Jaffe's method in an automated chemistry analyzer [9], using an isotope dilution mass spectrometry traceable calibrator. The estimated glomerular filtration rate (eGFR) was calculated using the Modification of Diet in Renal Diseases (MDRD) formula [10]. All the participants in the study were chosen only if they had an eGFR of more than 90 mL/min. It was done to ensure that participants had normal functioning kidneys, thereby preventing false elevations in galectin-3 levels. BMI was calculated for the participants using the formula, weight in kilograms divided by height in meter squares. Plasma galectin-3 was estimated by the ELISA method (R&D systems) using an ELISA plate analyzer and ELISA plate washer (Robonik, Ambernath, India).

Statistical analysis was done using IBM SPSS Statistics for Windows, Version 25.0. (IBM Corp., Armonk, NY). The percentage was used to compare the characteristics of participants in both the study groups with respect to the following factors: gender, history of smoking and alcohol intake, presence of diabetes mellitus, or hypertension. Mean and SD were calculated for BMI, LVEF, eGFR, serum creatinine, and plasma galectin-3. The student's t-test was used to compare galectin-3 levels between CHF patients and the controls. ANOVA was employed to compare galectin-3 levels in the different sub-groups of age, gender, and LVEF. The ROC curve was plotted for plasma galectin-3.

## Results

In the present study, the mean age of CHF patients was 55.9±8.1 years and 54.1±9.4 years for controls. Males constituted 63.6% (n=35) and females were 36.4% (n=20) in the CHF group, while 67.3% (n=37) were males and 32.7% (n=18) were females in the control group.

Table 1 depicts the characteristics of CHF patients and the controls. A total of 58.2% and 49.1% of the cases had a history of smoking and alcohol intake. A total of 70.9% of CHF patients had diabetes mellitus, while 83.6% were hypertensives. In the control group, 40% and 43.6% had a history of smoking and alcohol intake. A total of 40% and 54.4% of the controls had diabetes mellitus and hypertension, respectively. The mean and SDs for BMI, LVEF, eGFR, serum creatinine, and plasma galectin-3 levels are also depicted. The mean and SD for galectin-3 was 9.95±2.8 ng/mL and 4.08±1.3 ng/mL among cases and controls, respectively, with a p-value of 0.0001.

**Table 1:** Characteristics of chronic heart failure patients and the controls.

	Chronic heart failure patients (n=55)		Controls (n=55)	
	N	%	N	%
History of smoking	32	58.2	22	40
History of alcohol intake	27	49.1	24	43.6
Presence of diabetes mellitus	39	70.9	22	40
Presence of hypertension	46	83.6	30	54.5
	Mean	SD	Mean	SD
BMI	27.3	1.37	26.7	0.93
Left ventricular ejection fraction	35.4	4.6	68.84	5.64
Serum creatinine	0.75	0.09	0.69	0.10
Estimated glomerular filteration rate	100.55	8.5	111.27	12.3
Plasma galectin-3 (ng/mL)	9.95	2.8	4.08	1.3

Table 2 depicts the levels of plasma galectin-3 in age sub-groups and gender. As the age increased, levels of plasma galectin-3 increased in cases and controls, which was statistically significant. However, there was no statistical significance for levels of galectin-3 among males and females in both groups.

**Table 2:** Levels of plasma galectin-3 in age sub-groups and gender. N = number of participants. p-value significant at <0.01 (age sub-class) and <0.05 (gender sub-class).

	Galectin-3 (ng/mL) levels in chronic heart failure patients			Galectin-3 (ng/mL) levels in controls			
Age (in years)	N	Mean	SD	N	Mean	SD	p-value
41-50	18	7.45	0.75	24	3.2	0.7	<0.00001
51-60	20	9.33	1.14	16	4.47	1.1	
>60	17	12.9	2.1	15	5.27	0.704	
	Galectin-3 (ng/mL) levels in cases			Galectin-3 (ng/mL) levels in controls			
Gender	N	Mean	SD	n	Mean	SD	p-value
Male	35	9.5	3.04	37	4.1	1.39	0.403
Female	20	10.5	2.37	18	3.9	1.24	

Table 3 depicts the plasma galectin-3 levels in cases and controls when their LVEF was sub-classified. Percentage of LVEF was sub-grouped as 25-30, 31-35, 36-40, 41-45 in CHF patients and 56-60, 61-65, 66-70, >70 in the controls. There was a highly significant difference in galectin-3 levels between cases and controls when they were classified into sub-groups based on LVEF (p<0.0001).

**Table 3:** Levels of plasma galectin-3 in sub-groups of LVEF in CHF patients and controls. N: Number of participants; LVEF: Left ventricular ejection fraction; CHF: Chronic heart failure. P-value is significant at <0.05.

	Chronic heart failure patients				Controls			f-statistics	p-value
LVEF (%)	N	Mean	SD	LVEF (%)	N	Mean	SD	25.88	<0.0001
25-30	13	10.34	2.8	56-60	3	3.7	0.58		
31-35	12	9.1	1.5	61-65	13	5	0.79		
36-40	24	10.4	3.02	66-70	17	4	1.05		
41-45	6	8.7	3.9	>70	22	3.5	1.5		

**Figure 1:**
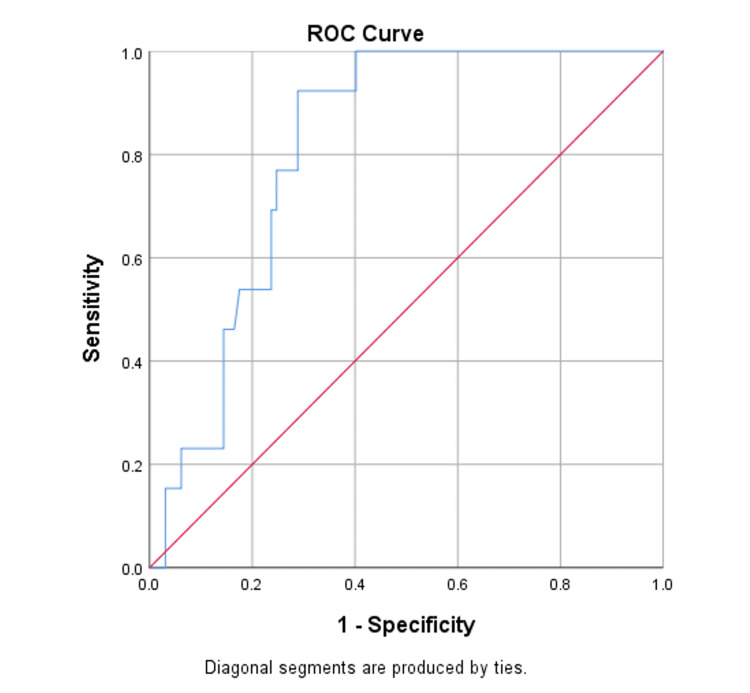
Receiver operating characteristic curve for plasma galectin-3 in chronic heart failure. Area under the curve: 0.813, cut-off value: 8 ng/mL.

Figure 1 depicts the diagnostic value of plasma galectin-3 by using the ROC curve. The ROC analysis was carried out using galectin-3 as the test variable and LVEF as an outcome. The area under the curve was found to be 0.813 with a statistically significant p-value <0.0001. The cut-off for galectin-3 is 8 ng/mL. Galectin-3 had a 92% sensitivity and specificity of 71% in predicting CHF. Hence, patients with galectin-3 more than 8 ng/mL are found to have more risk for CHF than galectin-3 cut-off less than 8 ng/mL.

## Discussion

HF is the final manifestation of all cardiovascular disorders. Multiple factors are involved in its evolution. Galectin-3 may be involved in the pathogenesis of HF. It has an advantage over the presently used markers as it does not vary with sudden hemodynamic changes [11]. The present study estimated the levels of plasma galectin-3 in CHF patients and controls. With respect to the participants' profile, cases and controls were matched for variables like age, gender, history of smoking and alcohol intake, presence of diabetes mellitus or hypertension, BMI, and eGFR.

In the present study, plasma galectin-3 levels were markedly increased in HF than in the controls. Similar results were reported by Shi Y et al. [12] and Jiang J et al. [13]. Further, in this study, the levels of galectin-3 increased significantly as age increased. Similar observations were documented by Krintus M et al. and Tang WH et al. with respect to serum galectin-3 and age sub-groups [14, 15]. Finally, in the present study, galectin-3 levels had no significance with gender. However, de Boer RA et al. reported that higher serum galectin-3 concentration was observed in women compared to the men in their study population [16].

The ROC curve in the present study revealed that plasma galectin-3 was 92% sensitive and 71% specific for diagnosing CHF. Moreover, as LVEF increased, the concentration of galectin-3 decreased, considering the cut-off value of 8 ng/mL. Galectin-3 levels in HF patients increased as their heart functioning worsened.

Jiang J et al. have reported that galectin-3 more than 15.9 ng/mL identified HF with preserved ejection fraction with 76% sensitivity and 72% specificity [13]. Daniels LB et al. [17] and Chen A et al. [18] have reported the prognostic importance of galectin-3 in the general population, independent of the established and frequently used marker, B-type natriuretic peptide (BNP)/NT pro-BNP. Amin HZ et al. have suggested that galectin-3 serves as an early mediator of fibrosis in the heart [11]. The FDA approved galectin-3 in 2010 for the assessment of HF together with clinical examination [19]. Galectin-3 has been identified as a novel marker reflecting fibrosis and would help in predicting adverse outcomes [19]. Shah RV et al. [20] and Djoussé L et al. [21] opined that galectin-3 measurement in HF patients provides additional diagnostic information and is also used to predict their prognosis.

Thus, galectin-3 as a biomarker will help identify the sub-set of CHF patients in whom maladaptive remodeling is the predominant pathogenesis pathway. This, in turn, will aid in effectively reversing adverse cardiac remodeling by designing drugs antagonizing the effects of galectin-3.

Limitations

The present study was done for a period of 10 months only using a small number of participants. Different cardiologists performed echocardiography on different patients, so the subjective variation in LVEF determination could not be eliminated. Correlating galectin-3 levels with BNP levels (an established marker) was not considered in the present study.

## Conclusions

Galectin-3 has a distinct role in the pathogenesis of CHF. It is proven to be involved in the process of left ventricular remodeling. This study has also evidenced an increase in galectin-3 levels in the plasma of CHF patients when compared to the control group. Hence, higher concentrations of galectin-3 are predictive of CHF.

Investigating galectin-3 levels during suspected CHF may have a role in early diagnosis, helping the physicians to achieve better therapeutic targets. Thus, it will improve the disease compliance of the patients and a future marker of research aiding disease-modifying therapy in CHF.
